# The effect of prior thecal puncture on cerebrospinal fluid analytes in normal adult horses

**DOI:** 10.1111/jvim.15842

**Published:** 2020-07-02

**Authors:** Hayley Chidlow, Steeve Giguère, Melinda Camus, Bridgette Wells, Roy Berghaus, Erin McConachie Beasley

**Affiliations:** ^1^ Department of Large Animal Medicine and Surgery University of Georgia College of Veterinary Medicine Athens Georgia USA; ^2^ Department of Pathology University of Georgia College of Veterinary Medicine Athens Georgia USA; ^3^ Department of Population Health University of Georgia College of Veterinary Medicine Athens Georgia USA

**Keywords:** equine, neurologic disease, repeat CSF collection

## Abstract

**Background:**

Serial cerebrospinal fluid (CSF) analysis might be required in clinical neurologic disease. The effect of lumbosacral (LS) or cervical (C1‐C2) centesis on subsequent CSF cytologic analyses has not been investigated in horses.

**Objective:**

To evaluate the effect of thecal puncture on subsequent CSF analyses

**Animals:**

Ten healthy adult horses.

**Methods:**

Prospective study. Horses were randomly assigned to undergo CSF collection twice, 14 days apart, from either the C1‐C2 or LS space. After a 4‐month washout period, CSF collection was repeated from the alternate site. Continuous data were analyzed using linear mixed‐effects models and count data using mixed‐effects negative binomial regression. Statistical significance was set at *P* < .05.

**Results:**

There was no significant effect of collection day (day 0 or day 14) for any CSF analytes, including protein concentration (C1‐C2: 45 [95% CI: 33‐57] mg/dL day 0 vs 49 [95% CI: 39‐62] mg/dL day 14, *P* = .12; LS: 64 [95% CI: 41‐100] mg/dL day 0 vs 83 [95% CI: 53‐129] mg/dL day 14, *P* = .37), or nucleated cell count (C1‐C2: 2 [95% CI: 1‐4] cells/μL day 0 vs 3 [95% CI: 1‐4] cells/μL day 14, *P* = .65; LS: 3 [95% CI: 2‐5] cells/μL day 0 vs 5 [95% CI: 3‐8] cells/μL day 14, *P* = .10). There was no significant difference in EPM titer or EPM serum : CSF ratio between days 0 and 14.

**Conclusions and Clinical Importance:**

Repeat thecal puncture from the LS or C1‐C2 space 2 weeks apart does not appear to impact CSF analytes.

**Abbreviations**
AI: antibody index
CSF: cerebrospinal fluid
EPM: equine protozoal myeloencephalitis
LS: lumbosacral
RBC: red blood cell
WBC: white blood cell

## 1 INTRODUCTION

Complete neurologic system evaluation and diagnosis of neurologic disease requires evaluation of cerebrospinal fluid (CSF). In many cases of equine neurologic disease, a single spinal centesis is performed, however there are occasions where repeat CSF analysis is warranted. Clinical scenarios in which this becomes relevant include when (a) clinical signs have worsened despite treatment, (b) repeat diagnostics are needed to determine duration of treatment or confirm initial results, or (c) for research purposes. Repeat CSF collection is performed regularly in human medicine, particularly in cases of bacterial meningitis.^1^, ^2^ In one human study, a small increase in total nucleated cell count (TNCC) was observed after repeat lumbar puncture 14 days apart.^3^ The effect of repeat thecal puncture on CSF indices (albumin quotient and IgG index) has been investigated in normal horses and those with equine protozoal myeloencephalitis (EPM).^4^, ^5^ After cervical canal endoscopy, mild inflammation (increased CSF protein and TNCC) was observed on day 7 and returned to near normal values on day 21.^6^ However, to the authors' knowledge, the effect of previous CSF collection from the lumbosacral (LS) or C1‐C2 space on subsequent CSF cytologic analyses, including TNCC, red blood cell (RBC) count, and protein concentration, has not been investigated.

The objective of our study was to evaluate the effect of prior thecal puncture on subsequent CSF analyses in horses. The authors' hypothesized that there would be no clinically relevant difference in analyses of CSF collected 14 days after initial thecal puncture.

## 2 MATERIALS AND METHODS

### 2.1 Animals

All experimental procedures were approved by the University of Georgia Institutional Animal Care and Use Committee. The study population consisted of 10 adult Quarter Horses, aged 3‐22 years. There were 4 mares, 4 geldings, and 2 stallions. All horses belonged to the University of Georgia research herd. Before enrollment, horses were determined to be healthy based on normal physical and neurologic examinations. The horses were housed in individual box stalls for 12 hours before and 48 hours after CSF collection. The horses were otherwise housed on pasture with access to shelter. Ad libitum water and coastal Bermuda hay were available throughout the study period.

### 2.2 Cerebrospinal fluid collection

Horses were randomly assigned to undergo CSF collection from either the C1‐C2 or LS space. An online random number generator (https://www.randomizer.org/) was used to determine which site was sampled first. CSF collection from the same site was repeated 14 days later. After a 4‐month washout period, physical and neurological examinations were repeated, and the groups were switched to undergo CSF collection from the alternative site. CSF collection from that same site was then repeated 14 days later.

Lumbosacral and cervical centesis were performed in standing, sedated horses as previously described.^7^, ^8^ Horses were restrained in stocks for CSF collection, and sedated with romifidine (80 μg/kg, IV; Sedivet, Boehringer Ingelheim Vetmedica, Duluth, GA) or detomidine hydrochloride (10 μg/kg, IV; Dormosedan, Zoetis, Parsippany, NJ). Lidocaine hydrochloride (100 mg; 2%; VetOne, Boise, ID) was infiltrated SC and into the musculature at both C1‐C2 and LS sites to provide local anesthesia. A 3.5 in. or 8 in. 18 gauge spinal needle (Mila International, Florence, KY) was used for cervical and LS centesis respectively. If more than 4 attempts were required for CSF collection, a new needle was used. A total of 5 mL of CSF was collected in 1 mL aliquots during each procedure, using 5 mL syringes, and placed into serum tubes. After collection of CSF, horses were administered flunixin meglumine (1.1 mg/kg IV; Banamine, Merck Animal Health, Madison, NJ). Whole blood (10 mL) was collected into a serum tube by jugular venipuncture. The blood was allowed to clot at room temperature, centrifuged at 3000 rpm for 15 minutes and the serum separated and frozen at −80°C until analysis.

### 2.3 Cerebrospinal fluid analysis

The third aliquot of CSF was submitted for fluid analysis (TNCC, RBC count, total protein concentration and cytospin for cytologic analysis) within 30 minutes of collection. If 3 aliquots could not be collected the second 1 mL aliquot was submitted. Manual TNCC and RBC count were performed on each sample using a standard Neubauer hemocytometer. Total protein concentration was quantitated via the biuret method (Cobas 6000 c 501; Roche Diagnostics, Basel, Switzerland). Cytologic evaluation was performed in batches by a clinical pathology resident (Bridgette Wells) and board‐certified clinical pathologist (Melinda Camus).

### 2.4 Equine protozoal myeloencephalitis cerebrospinal fluid titers

Serum and CSF from each horse was stored at −80°C and shipped to Equine Diagnostic Solutions Laboratory (Lexington, KY) for batched analysis within 6 months of collection. An enzyme linked immunosorbent assay to detect antibodies to *Sarcocystis neurona* (surface antigens 2, 4, and 3) was performed.^9^, ^10^

### 2.5 Statistical methods

Sample size calculation using published nucleated cell counts and protein concentrations measured from CSF collected from the LS space in horses^11^ indicated 10 horses would be sufficient to detect a 60% difference between sample times as being statistically significant with 80% power and *α* = .05. Normality of the data was assessed based on examination of histograms and normal Q‐Q plots of the residuals. Homogeneity of variance was assessed by plotting residuals against predicted values. Data were transformed to the natural logarithm when necessary. Protein concentration, RBC count, EPM titer in CSF, and serum : CSF titer ratios were assessed using linear mixed‐effects models with anatomic site and collection day modeled as fixed nominal effects and horse included as a random effect to account for repeated measurements. EPM titers reported as <2.5 in CSF samples or as <250 in serum samples were arbitrarily assigned values of 1.25 and 125, respectively, for statistical analysis. Samples with a CSF EPM titer <2.5 were considered to have a negative serum : CSF ratio, and were arbitrarily assigned a serum : CSF ratio of 400 for statistical comparisons. Counts of TNCC were fitted to a mixed‐effects negative binomial model. Percentages of neutrophils, lymphocytes and large mononuclear cells were analyzed using fractional probit regression with robust standard errors clustered on horse to account for the correlated structure of the data. Results of statistical models were reported using estimated marginal means to ensure that the effect of each variable was adjusted for other variables included in the model. For all analyses, model fit was assessed using Akaike's information criterion values. Values of *P* < .05 were considered statistically significant. Analyses were performed using commercially available statistical software (Stata version 16.1, StataCorp LLC, College Station, TX).

## 3 RESULTS

After randomization, 7 horses were assigned to undergo LS centesis first and 3 horses to undergo C1‐C2 centesis. After a 4‐month washout period, groups were switched, and horses underwent CSF collection from the second site. One horse was euthanized, for reasons unrelated to the study, during the washout period after undergoing LS centesis. Therefore, 10 horses underwent 2 LS centeses, and 9 horses underwent repeat cervical centesis, resulting in comparison of 19 CSF samples for day 0 and day 14.

CSF analytes are summarized by collection day and anatomic site in Table 1. There was no significant effect of collection day (day 0 or 14) for any analytes. CSF TNCC count was above the reference interval (RI: 0‐5 cells/μL)^7^, ^12^ in 4/19 (3 LS; 1 C1‐C2) day 0 samples and 5/19 (3 LS; 2 C1‐C2) day 14 samples (Table SS1). Substantial blood contamination of CSF (≥9000 RBC/μL) was present in 2/4 day 0 samples and 3/5 day 14 samples that had TNCC counts above the reference interval.

**TABLE 1:** Estimated marginal means (95% confidence intervals) for cerebrospinal fluid analytes collected on days 0 and 14 from cervical (C1‐2) and lumbosacral (LS) anatomic sites in 10 adult horses

Analyte	Site	Day 0	Day 14	*P* ^a^
Protein (mg/dL)^b^	C1‐2	45 (35, 57)	49 (39, 62)	.12
	LS	64 (41, 100)	83 (53, 129)	.37
WBC count (cells/μL)	C1‐2	2 (1, 4)	3 (1, 4)	.65
	LS	3 (2, 5)	5 (3, 8)	.10
RBC count (cells/μL)^b^	C1‐2	20 (1, 240)	75 (6, 774)	.24
	LS	38 (2, 493)	145 (10, 1860)	.36
% Neutrophils	C1‐2	14 (0, 32)	19 (0, 38)	.47
	LS	6 (0, 16)	20 (4, 37)	.16
% Lymphocytes	C1‐2	75 (54, 96)	76 (53, 98)	.58
	LS	81 (70, 92)	72 (57, 86)	.34
% large mononuclear cells	C1‐2	10 (0, 24)	2 (0, 4)	.25
	LS	12 (5, 19)	8 (3, 12)	.28
EPM titer^b^	C1‐2	2 (1, 2)	2 (1, 3)	.37
	LS	2 (1, 3)	3 (2, 5)	.13
Serum : CSF EPM titer ratio^b^	C1‐2	281 (187, 421)	260 (173, 390)	.37
	LS	264 (135, 516)	162 (83, 317)	.26

*Note:* There was a 4‐month washout period between sample collections at the different anatomic sites.

^a^ Contrast of day marginal means.

^b^ Geometric means back transformed from the logarithmic scale.

The median number of collection attempts required to obtain 38 CSF samples was 2 (range 1‐10). For cervical centesis (18 samples), a median of 1 attempt was required (range 1‐5). For LS centesis (20 samples), a median of 3 attempts were required (range 1‐10). In horses with substantial blood contamination (≥9000 RBC/μL), an average of 2 collection attempts (range 1‐3) were required for both cervical and LS centesis.

For samples collected from the C1‐C2 space, 1/9 horses had a higher CSF EPM titer on day 14 compared to day 0, and 8/9 horses had the same titer on both days (Table SS2). All horses had a serum : CSF titer ratio ≥ 100 at both day 0 and day 14, and were classified as EPM negative using a cutoff of ≤50. This cutoff was chosen as it provides increased specificity compared to a cutoff of ≤100, and is therefore more appropriate as a screening test in this population of horses without signs of neurologic disease.^9^

For samples collected from the LS space, 5/10 horses had a higher CSF EPM titer on day 14 compared to day 0, 1/10 horses had a lower titer, and 4/10 horses had the same titer on both days. No horses had a serum : CSF titer ratio ≤ 50 on day 0. On day 14, 2/10 horses transitioned from a negative to a positive result (serum : CSF titer ratio of 50). One horse (EPM CSF titer of 5) had substantial blood contamination of CSF (51 900 RBC/uL) at day 14. The other horse (CSF EPM titer of 10) had minimal blood contamination of CSF at either day 0 or day 14, however CSF protein concentration was markedly increased at day 14 (257 mg/dL) compared to day 0 (53 mg/dL). Antibody indices for both horses were below 1 (0.21 and 0.38, respectively), therefore they were classified as EPM negative despite the serum : CSF titer ratio.^13^

After CSF collection, no abnormalities in physical or neurologic examinations were noted. No gross inflammation was observed at either the LS or C1‐C2 centesis sites after CSF collection on day 0 or day 14.

## 4 DISCUSSION

Based on the results of our study, recent thecal puncture does not appear to affect subsequent CSF analysis to a degree that would interfere with clinical interpretation of results.

In human medicine, a small increase in TNCC, neutrophil count, and mononuclear cell count was identified in healthy adults undergoing repeat lumbar puncture, and 56% of these patients met the definition for CSF pleocytosis on day 14.^3^ In the present study, there was no statistical difference between CSF collected on day 0 compared to day 14. Two horses had mild CSF pleocytosis in the absence of blood contamination on day 14 but not day 0 (Table SS1). Two horses had mild CSF pleocytosis on both day 0 and day 14; in both cases there was substantial blood contamination (≥35 000 RBC/μL) on day 14 only. Two horses had CSF pleocytosis on day 0 only; blood contamination (≥9000 RBC/μL) was present in both. While CSF pleocytosis without blood contamination on day 14 could reflect inflammation induced by prior CSF collection, care must be taken not to overinterpret these results in light of the equivalent finding on day 0. Given the small increase in TNCC above the reference interval and lack of clinical signs of neurological disease or pain at the centesis site, the finding of CSF pleocytosis at day 14 is unlikely to be clinically relevant and more likely represents individual variation.

Historically, formulas have been used to correct TNCC and protein concentration in CSF with blood contamination, however this has now been shown to be inaccurate.^11^, ^14^, ^15^ Sequential CSF collection has been shown to decrease RBC contamination and associated increased TNCC and protein concentration. Nucleated cell count was shown to be significantly associated with RBC count in initial samples collected, with a minimum of 3 samples recommended to decrease iatrogenic blood contamination.^11^ In all cases with visible blood contamination at day 14, only 2 sequential samples of CSF were able to be obtained; the increased TNCC observed in these horses is presumed to be associated with blood contamination rather than because of inflammation associated with the previous CSF collection.

A primary reason for repeat thecal puncture in horses is in cases of suspected EPM with acute onset of clinical signs where initial diagnostic testing is negative. Neurologic signs can be observed before seroconversion in recently infected horses, yielding false negative results.^10^ If initial EPM testing is negative but clinical suspicion of EPM is high, repeat CSF collection could be indicated. Two horses in our study had positive serum : CSF titer ratios at day 14 from the LS space, compared to negative ratios at day 0. The CSF antibody titers in these horses were 5 and 10; the likelihood ratio for EPM of these titers are 0.083 and 0.74, respectively.^9^ The antibody index (AI) was also <1 in both cases, indicating the CSF *S neurona* antibody titer was of extraneural origin. The horses were therefore considered EPM negative.^13^ In 1 horse (CSF titer 5, AI .21), substantial blood contamination of CSF occurred (RBC 51 900 cells/μL), and CSF albumin concentration was increased to 331 mg/dL, therefore the change in CSF titer between day 0 and day 14 and subsequent positive ratio is presumed secondary to a traumatic tap and blood contamination. The second horse (CSF titer 10, AI 0.38), had minimal blood contamination (RBC 13 cells/μL), however CSF albumin concentration was increased to 198 mg/dL. The low AI is consistent with passive diffusion of antibody across the blood‐brain barrier rather than intrathecal antibody production. Although there was little evidence of a traumatic tap (minimal blood contamination, 1 attempt for CSF collection) on either day 0 or day 14, the authors consider this the likely cause of increased CSF albumin. In retrospect, the authors could have considered measuring serum albumin concentration to calculate albumin quotient, an indicator of blood‐brain barrier dysfunction.^16^

The most common reason for repeat thecal puncture in human medicine is in cases of bacterial meningitis that fail to improve with antimicrobial treatment. Bacterial meningitis is rare in adult horses but occurs more commonly in foals. Other indications in human medicine include reducing intracranial pressure, monitoring response to treatment, or investigating patients with persistent or relapsing fever.^1^ In equine medicine, other rationales include clinical research, including investigation of neuroaxonal dystrophy and other vitamin E responsive diseases.^17^ Serial CSF analysis could also play a role in investigation of poorly understood diseases such as neuroborreliosis.

A 14 day time period between CSF collection was chosen. This time period is (a) sufficient to allow seroconversion in horses recently infected with EPM^10^ and (b) considered by the authors to be an appropriate duration of time for initial antimicrobial treatment of bacterial meningitis before repeating CSF collection to assess treatment response.

One limitation of our study is the small sample size. The number of horses enrolled was based on power calculations to detect a statistical difference based on TNCC and protein concentrations, and was limited by availability of horses from the University of Georgia research herd. Because of the extended washout period between CSF collection from the C1‐C2 and LS sites, only university‐owned animals were used to avoid cost‐prohibitive fees associated with boarding donation horses.

In conclusion, this study suggests that repeat thecal puncture from the LS or C1‐C2 space 2 weeks apart does not result in clinically important changes in CSF analytes including total protein concentration, TNCC and RBC count. The difference in EPM serum : CSF titer ratio between day 0 and day 14 observed in 2 horses was unexpected, however AI was not consistent with intrathecal antibody production, and would not have led to a false positive diagnostic test result for EPM. In practice, a clinically important difference in CSF analytes is not expected from thecal puncture performed 14 days earlier.

## CONFLICT OF INTEREST DECLARATION

Authors declare no conflict of interest.

## OFF‐LABEL ANTIMICROBIAL DECLARATION

Authors declare no off‐label use of antimicrobials.

## INSTITUTIONAL ANIMAL CARE AND USE COMMITTEE (IACUC) OR OTHER APPROVAL DECLARATION

This study was approved by the University of Georgia IACUC.

## HUMAN ETHICS APPROVAL DECLARATION

Authors declare human ethics approval was not needed for this study.

## Supporting information


**Table S1** Cerebrospinal fluid red blood cell (RBC) count, white blood cell (WBC) count, and total protein concentration from individual horses from both the cervical (C1‐2) and lumbosacral (LS) space on day 0 and day 14.

**Table S2** Serum and cerebrospinal fluid equine protozoal myeloencephalitis (EPM) titers and serum : CSF titer ratios from individual horses from both the cervical (C1‐2) and lumbosacral (LS) space on day 0 and day 14.
