# Supplementary material for: The effect of prior thecal puncture on cerebrospinal fluid analytes in normal adult horses
Source: J Vet Intern Med. 2020 Jul 2;34(5):2117–21. doi: 10.1111/jvim.15842 (PMC7517835; doi:10.1111/jvim.15842)
Supplement: Supplementary file 1 — Table S1 Cerebrospinal fluid red blood cell (RBC) count, white blood cell (WBC) count, and total protein concentration from individual horses from both the cervical (C1‐2) and lumbosacral (LS) space on day 0 and day 14. [file JVIM-34-2117-s001.pdf]

Supplementary Table 1. Cerebrospinal fluid red blood cell (RBC) count, white blood cell (WBC) count, and total protein concentration from individual horses from both the cervical (C1-2) and lumbosacral (LS) space on Day 0 and Day 14.

		WBC (cells/uL)		RBC (cells/uL)		Protein (mg/dL)		CSF Appearance	
Horse	Site	Day 0	Day 14	Day 0	Day 14	Day 0	Day 14	Day 0	Day 14
1	C1-2	1	1	20	1	48	46	Colorless	Colorless
	LS	2	4	3	13	53	257	Colorless	Colorless
2	C1-2	2	1	1	0	87	102	Colorless	Colorless
	LS	2	2	0	1	85	97	Colorless	Colorless
3	C1-2	n/a	n/a	n/a	n/a	n/a	n/a	n/a	n/a
	LS	1	7	4	0	48	56	Colorless	Colorless
4	C1-2	1	9	3	10	50	62	Colorless	Colorless
	LS	4	4	0	7	72	75	Colorless	Colorless
5	C1-2	8	4	9000	1900	39	33	Blood-tinged	Colorless
	LS	6	7	228	35400	33	119	Colorless	Blood-tinged
6	C1-2	2	1	2	7	44	46	Colorless	Colorless
	LS	7	2	32200	1150	306	53	Colorless	Colorless
7	C1-2	2	1	29	57	37	41	Colorless	Colorless
	LS	1	4	911	124	52	73	Blood-tinged	Colorless
8	C1-2	2	6	820	18500	29	35	Colorless	Blood-tinged
	LS	8	17	8	51900	29	74	Colorless	Blood-tinged
9	C1-2	1	1	61	4480	40	53	Colorless	Colorless
	LS	1	4	194	83	78	53	Colorless	Colorless
10	C1-2	1	0	0	446	57	56	Colorless	Colorless
	LS	2	3	31	866	63	83	Colorless	Colorless

n/a = Not applicable because the sample was not collected.
