# Supplementary material for: The effect of prior thecal puncture on cerebrospinal fluid analytes in normal adult horses
Source: J Vet Intern Med. 2020 Jul 2;34(5):2117–21. doi: 10.1111/jvim.15842 (PMC7517835; doi:10.1111/jvim.15842)
Supplement: Supplementary file 2 — Table S2 Serum and cerebrospinal fluid equine protozoal myeloencephalitis (EPM) titers and serum : CSF titer ratios from individual horses from both the cervical (C1‐2) and lumbosacral (LS) space on day 0 and day 14. [file JVIM-34-2117-s002.pdf]

Supplementary Table 2. Serum and cerebrospinal fluid equine protozoal myeloencephalitis (EPM) titers and serum:CSF titer ratios from individual horses from both the cervical (C1-2) and lumbosacral (LS) space on Day 0 and Day 14.

		EPM Serum Titer		EPM CSF Titer		Serum / CSF Titer Ratio	
Horse	Site	Day 0	Day 14	Day 0	Day 14	Day 0	Day 14
1	C1-2	500	500	2.5	2.5	200	200
	LS	500	500	2.5	10[a]	200	50
2	C1-2	250	250	2.5	2.5	100	100
	LS	250	250	<2.5	<2.5	Negative	Negative
3	C1-2	n/a	n/a	n/a	n/a	n/a	n/a
	LS	250	250	<2.5	<2.5	Negative	Negative
4	C1-2	500	500	2.5	5	200	100
	LS	500	500	2.5	5	200	100
5	C1-2	250	250	<2.5	<2.5	Negative	Negative
	LS	500	500	<2.5	5[b]	Negative	100
6	C1-2	500	500	2.5	2.5	200	200
	LS	250	500	2.5	5	100	100
7	C1-2	250	250	<2.5	<2.5	Negative	Negative
	LS	500	500	5	2.5	100	200
8	C1-2	500	250	<2.5	<2.5	Negative	Negative
	LS	250	250	<2.5	5[c]	Negative	50
9	C1-2	<250	<250	<2.5	<2.5	Negative	Negative
	LS	<250	<250	<2.5	<2.5	Negative	Negative
10	C1-2	500	250	<2.5	<2.5	Negative	Negative
	LS	500	500	<2.5	2.5	Negative	Negative

n/a = Not applicable because the sample was not collected.
[a] CSF albumin increased (198.6mg/dL); Specific Index 0.38
[b] CSF albumin increased (169.3mg/dL); Specific Index 0.19
[c] CSF albumin increased (331.9mg/dL); Specific Index 0.21
